# Association of family history of cardiovascular disease with the prevalence of cardiometabolic risk factors in young adults in the United Arab Emirates: The UAE healthy future study

**DOI:** 10.1371/journal.pone.0319648

**Published:** 2025-03-12

**Authors:** Fatima Mezhal, Amar Ahmad, Abdishakur Abdulle, Andrea Leinberger-Jabari, Abdulla AlJunaibi, Abdulla Alnaeemi, Ayesha S. Al Dhaheri, Eiman AlZaabi, Fatma Al-Maskari, Fatme AlAnouti, Juma Alkaabi, Marina Kazim, Mohammad Al-Houqani, Mohammad Hag Ali, Naima Oumeziane, Omar El-Shahawy, Scott Sherman, Syed M. Shah, Tom Loney, Wael Almahmeed, Youssef Idaghdour, Luai A. Ahmed, Raghib Ali

**Affiliations:** 1 Public Health Research Center, New York University Abu Dhabi, Abu Dhabi, United Arab Emirates; 2 Department of Pediatric Endocrinology, Danat Al Emarat Hosiptal, Abu Dhabi, United Arab Emirates; 3 Department of Cardiology, Zayed Military Hospital, Abu Dhabi, United Arab Emirates; 4 Department of Nutrition and Health, College of Medicine and Health Sciences, UAE University, Al-Ain, United Arab Emirates; 5 Department of Pathology, Sheikh Shakhbout Medical City, Abu Dhabi, United Arab Emirates; 6 Institute of Public Health, College of Medicine and Health Sciences, UAE University, Al-Ain, United Arab Emirates; 7 Zayed Center for Health Sciences, UAE University, Al-Ain, United Arab Emirates; 8 College of Natural and Health Sciences, Zayed University, Abu Dhabi, United Arab Emirates; 9 Department of Internal Medicine, College of Medicine and Health Sciences, UAE University, Al-Ain, United Arab Emirates; 10 Abu Dhabi Blood Bank Services, SEHA, Abu Dhabi, United Arab Emirates; 11 Department of Medicine, College of Medicine and Health Sciences, UAE University, Al-Ain, United Arab Emirates; 12 Department of Health Science, Higher Colleges of Technology, Abu Dhabi, United Arab Emirates; 13 Department of Population Health, New York University School of Medicine, New York, New York, United States of America; 14 College of Medicine, Mohammed Bin Rashid University of Medicine and Health Sciences, Dubai, United Arab Emirates; 15 Heart and Vascular Institute, Cleveland Clinic Abu Dhabi, Abu Dhabi, United Arab Emirates; 16 MRC Epidemiology Unit, University of Cambridge, Cambridge, UK; University of Sharjah College of Health Sciences, UNITED ARAB EMIRATES

## Abstract

**Introduction:**

Family history of cardiovascular disease (CVD) is an independent risk factor for coronary heart disease, and the risk increases with number of family members affected. It offers insights into shared genetic, environmental and lifestyle factors that influence heart disease risk. In this study, we aimed to estimate the association of family history of CVD and its risk factors, as well as the number of affected parents or siblings, with the prevalence of major cardiometabolic risk factors (CRFs) such as hypertension, dysglycemia, dyslipidemia and obesity in a sample of young adults.

**Methods:**

The study utilized a cross-sectional analysis of baseline data from the UAE Healthy Future Study (UAEHFS), involving 5,058 respondents below the age of 40 years. Information on parental and sibling health regarding heart disease and stroke, hypertension, type 2 diabetes (T2D), high cholesterol and obesity, was gathered through a self-completed questionnaire. CRFs were estimated based on body measurements, biochemical markers and self-reported conditions. Multivariate regression analyses were used to examine the associations between categories of family history and the estimated CRFs.

**Results:**

More than half (58%) of the sample reported having a positive family history of CVD or its risk factors. The most common family history reported was T2D and hypertension, which accounted for 39.8% and 35% of the sample, respectively. The prevalence of all CRFs was significantly higher among those with a positive family history compared to those without family-history (P < 0.001). The prevalence and likelihood of having a CRF increased as the number of parents and/or siblings affected increased, indicating a potential dose-response trend. The odds were highest among individuals with both parental-and-sibling family history of disease, where they increased to 2.36 (95% CI 1.68-3.32) for hypertension, 2.59 (95% CI 1.86-3.60) for dysglycemia, 1.9 (95% CI 1.29-2.91) for dyslipidemia and 3.79 (95% CI 2.83-5.06) for obesity.

**Conclusion:**

In this study, we addressed the effect of family history as an independent risk factor on the major CRFs for the first time in the region. We observed that the majority of young Emirati adults had a positive family history of CVD-related diseases. Family history showed a strong association with the increased prevalence of CRFs. Additionally, having more relatives with specific diseases was associated with a higher risk of developing CRFs. Identifying people with a history of these conditions can help in early intervention and personalized risk assessments.

## Introduction

Family health history is an integral part of an individual’s health assessment, especially in the context of noncommunicable disease. It can represent the genetic susceptibility to a disease as well as the shared environmental and behavioral background. Individuals with a family history of cardiovascular disease (CVD), diabetes, or cancer have a two- to five-fold increase in their risk of developing these diseases, compared to someone without such a history [1]. Family history data are factored into multiple disease risk assessments to identify individuals at increased risk [2, 3].

In the context of cardiovascular disease, a family history of CVD is considered an independent risk factor for premature coronary heart disease in offspring, where it modifies future CVD risk depending on the number and age of those affected [4]. Reports from the Framingham cohort showed that offspring of parents with premature CVD have a 60-75% risk increase, while individuals with siblings with CVD have 40% increased risk [5]. A case-control study led by Chacko et al. [6] showed that the risk of premature coronary heart disease increased linearly with increasing number of affected family members. Another study on adolescents led by Silva et al. [7] showed that a family history of CVD was associated with 50%, 193% and 116% increase in the odds of high BMI, triglycerides, and fasting glucose, respectively. Most of these studies suggested that family history of CVD alone is generally sufficient to capture susceptibility to future CVD in offspring [2,8].

In addition to family history of CVD, it is also important to take into account the history of the main CVD riskfactors. Family history of hypertension, type 2 diabetes (T2D), dyslipidemia and obesity, can also have a significant impact on the cardiometabolic health of offspring. For example, data from the EPIC-InterACT study, a well-powered cohort study that focuses on genetic-and-lifestyle interplay, showed that a family history of diabetes in one parent increased the risk of diabetes by 2.44-fold, and the risk increased to 5.14-fold when both parents had a history of diabetes [9–11]. Another cross-sectional study on 5000 participants showed that having a family history of hypertension increased the risk of hypertension, obesity and metabolic syndrome in the offspring [12]. Additionally, obesity-focused studies have shown that having obese parents increased the odds of obesity in their offspring up to 22-fold, independent of age, sex, socioeconomic status and ethnicity [13].

According to the World Health Organization (WHO), CVD is attributable to 40% of the causes of deaths in the UAE [14]. Our recent reports showed that CRFs are highly prevalent, even in the younger age groups below the age of 40 years [15, 16]. This high prevalence is partially due to rapid urbanization, lifestyle changes, and genetic predisposition [17]. Family history information can give insight to how some of these factors can influence heart disease risk.

There are limited studies in the UAE that addressed family history of CVD as an independent risk factor for CVD [18, 19]. The past studies showed correlations between family history and obesity, but found limited associations with other CRFs. In this study, we sought to investigate the association of family history of CVD as well as history of main CVD- risk factors with the prevalence of CRFs in young Emirati adults. We also investigated the effect of number of parents and/or siblings affected with prevalence of CRFs in the sample.

## Materials and methods

### Study design and settings

The United Arab Emirates Healthy Future Study (UAEHFS) is a population-based cohort study that is designed to explore the risk factors for CVD in the Emirati population. The study invites Emirati volunteers in different cities in the UAE, in different settings such as health centers, universities and companies. Participants must be 18 and above, without acute illnesses or pregnancy, and able to provide an informed consent.

This study was based on cross-sectional analysis of the baseline data collected between February 2016 and December 2018. The study sample was retrieved from the UAE Healthy Future Study (UAEHFS) in February 2019 [20].

### Ethical statement

All participants provided informed written consent. This study followed the principles outlined in the Declaration of Helsinki and was approved by the Abu Dhabi Health Research and Technology Committee (ref. DOH/HQD/2020/516) and NYUAD’S Research Ethics Committee (REC) (ref. 0072017R). Additional information on the UAEHFS methodology is published elsewhere [20].

### Data collection

Study participants of the UAEHFS were asked to answer a self-completed questionnaire that was previously tested and validated [20]. The questionnaire collected sociodemographic, health, lifestyle, and family history data. The family history section collected data on maternal, paternal and sibling health status and if they were diagnosed with chronic diseases including heart disease and stroke, as well as the major CVD risk factors such as hypertension, diabetes, high cholesterol and/or obesity.

Participants then underwent physical measurements, including two repeat measures of brachial blood pressure and anthropometrics. Blood samples were collected to measure glycemic and lipid markers. All measurements and samples were taken using a consistent protocol to ensure data consistency and reliability, as detailed in other sources [20].

### Family history and cardiometabolic risk factors criteria

Data from the family history questionnaire were categorized based on the parental and sibling history of heart disease and stroke, hypertension, diabetes, high cholesterol and obesity. Data were sub-categorized based on number of first-degree relatives reporting having the disease; (0) no family history, (1) uni-parental (one parent reporting disease), (2) bi-parental (both parents reporting disease), (3) only sibling(s) without parents, and (4) any parent and siblings combined.

Hypertension was defined as having two consecutive blood pressure readings of ≥ 140 mmHg systolic and/or ≥ 90 mmHg diastolic [21]. Additionally, hypertension was also defined by a self-reported prior diagnosis or current use of blood pressure medication. Dysglycemia was defined as having an HbA1c ≥ 5.7%, fasting blood glucose ≥ 126 mg/dl, or reporting diabetes or taking antidiabetic medication [22, 23].

Dyslipidemia was defined as having an abnormal level of any of the following lipid markers: LDL cholesterol level of ≥ 130 mg/dL, HDL cholesterol level of ≤ 40 mg/dL for men or ≤ 50 mg/dL for women, total cholesterol ≥ 200 mg/dL, or triglycerides ≥ 150 mg/dL for fasting samples and ≥ 175 mg/dL for random (non-fasting) samples, in addition to self-reporting prior diagnosis of high cholesterol or taking lipid-controlling medication [24, 25]. Finally, obesity was defined as having a body mass index (BMI) ≥ 30.0 kg/m^2^. All CRFs were binarized to 0 and 1, indicating whether they have the CRF or not, based on the criteria mentioned.

### Statistical analysis

Categorical variables were summarized using frequency and percentage. A Pearson chi-square test was applied to investigate the association between family history (yes, no) and categorical variables, such as sex, behavioral risk factors and CRFs. Age was summarized as mean and standard deviation. Student’s t-test was used to compare the mean of age between the groups of positive family history and no family history.

In the first analysis, multivariate logistic regression models were performed to investigate the associations between family history of the following: heart disease/stroke, hypertension, type 2 diabetes, high cholesterol, and obesity as predictors. Each model included a specific outcome variable, such as hypertension, dysglycemia, dyslipidemia, and obesity, adjusted for age and gender. Subsequently, further multivariate logistic regression models were conducted as in the initial analysis, with each model additionally adjusted for age, sex, and other types of family history to assess the effect of specific family histories with respective disease

Odds ratios (ORs) and their corresponding 95% confidence interval (CI) were estimated for each predictor variable in the multivariate logistic regression models. To visually represent the relationships between the predictor variables and the outcomes, odds ratios and their corresponding 95% CIs were plotted. All applied tests were two sided. P-values less than 0.05 were considered as statistically significant. Statistical analyses were performed by STATA version 16.1 [26].

## Results

The UAEHFS population at the time of data access was 5,167 individuals. Respondents without family history data were excluded from the study, resulting in a final sample size of 5,058 participants. Among them, 57.6% had a positive family history of CVD. Table 1 provides an overview of the population characteristics. Approximately 62% of the study population were males, and 38% were females. The mean age of the sample was 25.7 (±6.1). Additionally, 25.7% were current smokers and 37.5% were classified as having a low level of physical activity.

**Table 1:** Characteristics of the study population.

	Total n = 5,058	No family history n = 2,147 (42.5%)	Positive family history n = 2,911 (57.6%)	P value
**Sex**:				<0.001
* Men*	3,136 (62.0%)	1,409 (65.6%)	1,727 (59.3%)	
* Women*	1,922 (38.0%)	738 (34.4%)	1,184 (40.7%)	
**Age:**	25.7 (±6.1)	24.8 (±5.9)	26.6 (±6.3)	<0.001
**Smoking status:**				0.622
* Non-smokers*	2,628 (52.0%)	797 (37.1%)	1,831 (62.9%)	
* Current smokers*	1,299 (25.7%)	384 (17.9%)	915 (31.4%)	
**Physical activity:**				0.126
* Moderate – high*	447 (8.8%)	112 (5.2%)	335 (11.5%)	
* Low*	1,894 (37.5%)	543 (25.3%)	1,351 (46.4%)	
**Cardiometabolic risk factors (CRFs):**				
* Hypertension*	1,098 (21.7%)	362 (16.9%)	736 (25.3%)	<0.001
* Dysglycemia*	635 (12.6%)	222 (10.3%)	413 (14.2%)	<0.001
* Dyslipidemia*	3,123 (61.7%)	1,241 (57.8%)	1,882 (64.7%)	<0.001
* Obesity*	1,308 (25.9%)	457 (21.3%)	851 (29.2%)	<0.001

Data is presented as n (%) or mean (±SD).

Family history is defined as having self-reported any first-degree (parental or sibling) family history of the following diseases (heart disease and/or stroke, hypertension, type 2 diabetes, high cholesterol, and/or obesity).

Total percentages include missing values (column%). Missing data (%) is calculated as 5.6% for hypertension, 0.2% for dysglycemia, 0.1% for dyslipidemia, 5.2% for obesity, 22.4% for smoking, and 53.7% for physical activity.

The cardiometabolic characteristics of the sample show that 21.7% had hypertension, 12.6% had dysglycemia, 61.7% had abnormal lipid markers and 25.9% were classified as obese. Individuals with a positive family history of CVD or any CVD- risk factor exhibited statistically significant higher proportions of CRFs compared to those without family history (p-values < 0.001) (Table 1).

### Family history of CVD and its risk factors and the prevalence of CRFs

The highest positive family history was observed for T2D (39.8%) and hypertension (35.0%), while the family history of heart disease and/or stroke was the lowest (16.2%). Table 2 shows the age and sex- adjusted prevalence of CRFs according to family history categories. The prevalence of all CRFs was higher in the categories of positive family history compared to those without family history (P < 0.001). Moreover, the proportions of CRFs increased as the number of relatives with the disease increased, as shown for hypertension, dysglycemia and obesity.

**Table 2:** Age- and gender- adjusted prevalence of cardiometabolic risk factors according to the study population’s family history levels.

		Prevalence of Cardiometabolic risk factors (CRFs) in the sample			
Family history of:	N (%)	Hypertension	Dysglycemia	Dyslipidemia	Obesity
**Heart disease and/or stroke**					
-(0) No FH	4,239 (83.8)	19.6 (18.3-20.9)	11.0 (10.0-11.9)	62.0 (60.5-63.6)	25.8 (24.4-27.2)
-Positive FH:	819 (16.2)				
-(1) Uniparental	673 (13.3)	25.4 (22.0-28.8)	15.5 (12.9-18.2)	66.5 (62.7-70.3)	30.0 (26.5-33.5)
-(2) Biparental	55 (1.1)	24.1 (12.8-35.3)	19.1 (9.5-28.7)	72.0 (58.8-85.2)	27.1 (15.6-38.6)
-(3) Sibling only	51 (1.0)	21.0 (9.4-32.5)	7.6 (0.3- 14.8)	65.2 (51.9-78.5)	28.2 (15.5-41.0)
-(4) Any parental + sibling	40 (0.8)	34.6 (19.4-49.8)	8.9 (0.1-16.6)	68.4 (52.6-84.3)	34.6 (19.8-49.5)
**Hypertension**					
-(0) No FH	3,293 (65.0)	17.2 (15.8-18.6)	10.9 (9.8-11.9)	61.2 (59.4-62.9)	24.9 (23.3-26.4)
-Positive FH:	1,765(35.0)				
-(1) Uniparental	1,121 (22.2)	23.2 (20.6-25.7)	11.6 (9.7 -13.5)	65.8 (62.9-68.6)	29.1 (26.431.8)
-(2) Biparental	392 (7.8)	30.5 (25.7-35.3)	15.0 (11.6-18.5)	64.8 (59.8-69.7)	26.1 (21.7-30.5)
-(3) Sibling only	44 (0.9)	36.5 (21.5-51.5)	19.8 (8.4-31.2)	76.4 (63.3-89.5)	33.2 (18.9-47.5)
-(4) Any parental + sibling	208 (4.1)	36.5 (29.5-43.5)	15.3 (10.7-19.9)	65.9 (59.0-72.9)	36.9 (30.2-43.6)
**Type 2 Diabetes**					
-(0) No FH	3,044 (60.2)	17.2 (15.7–18.6)	9.8 (8.7-10.9)	60.8 (58.9-62.6)	24.0 (22.4-25.6)
-Positive FH:	2,014 (39.8)				
-(1) Uniparental	1,215 (24.0)	25.3 (22.7-27.9)	12.0 (10.2-13.9)	66.3 (63.6-69.0)	28.0 (25.4-30.6)
-(2) Biparental	447 (8.8)	25.7 (21.6-29.9)	16.2 (12.9-19.5)	63.7 (59.0-68.4)	32.5 (28.1-37.0)
-(3) Sibling only	70 (1.4)	25.9 (15.3-36.6)	15.2 (7.0-23.4)	61.1 (49.2-73.0)	24.9 (14.9-34.9)
-(4) Any parental + sibling	282 (5.6)	25.9 (20.7-31.1)	21.5 (16.8-26.1)	68.8 (62.9-74.6)	37.1 (31.4-42.9)
**High cholesterol**					
-(0) No FH	3,797 (75.0)	19.6 (18.2-21.0)	11.3 (10.3-12.4)	61.3 (59.7-62.9)	26.3 (24.8-27.8)
-Positive FH:	1,261 (25.0)				
-(1) Uniparental	764 (15.1)	21.4 (18.4-24.4)	11.6 (9.4-13.9)	64.8 (61.3-68.2)	24.8 (21.7-27.9)
-(2) Biparental	267 (5.3)	21.0 (16.0-26.1)	13.3 (9.4-17.2)	68.1 (62.3-74.0)	28.4 (22.9-33.9)
-(3) Sibling only	63 (1.3)	30.4 (18.6-42.1)	18.8 (9.4-28.1)	67.9 (55.7-80.1)	31.4 (20.0-42.9)
-(4) Any parental + sibling	167 (3.3)	33.7 (26.0-41.4)	12.0 (7.4-16.7)	78.1 (71.5-84.7)	34.1 (26.8-41.4)
**Obesity**					
-(0) No FH	4,140 (81.9)	19.6 (18.2-20.9)	11.4 (10.4-12.4)	61.7 (60.2-63.3)	24.1 (22.7-25.4)
-Positive FH:	918 (18.1)				
-(1) Uniparental	414 (8.2)	21.2 (17.2-25.3)	13.1 (9.9-16.3)	63.9 (59.1-68.6)	27.2 (22.8-31.6)
-(2) Biparental	60 (1.2)	33.9 (21.0-46.9)	7.8 (1.1-14.4)	61.8 (49.1-74.5)	33.9 (21.5-46.3)
-(3) Sibling only	199 (3.9)	31.8 (24.7-38.9)	11.4 (6.9-15.8)	72.1 (65.9-78.4)	38.1 (31.1-45.1)
-(4) Any parental + sibling	245 (4.8)	23.6 (17.8-29.3)	13.8 (9.4-18.2)	71.9 (66.2-77.5)	53.5 (47.0 – 60.0)

Data is presented as N (%) and percentages (95% confidence intervals).

FH; Family history.

### Association between family history of CVD and its risk factors and the CRFs

**Fig 1:**
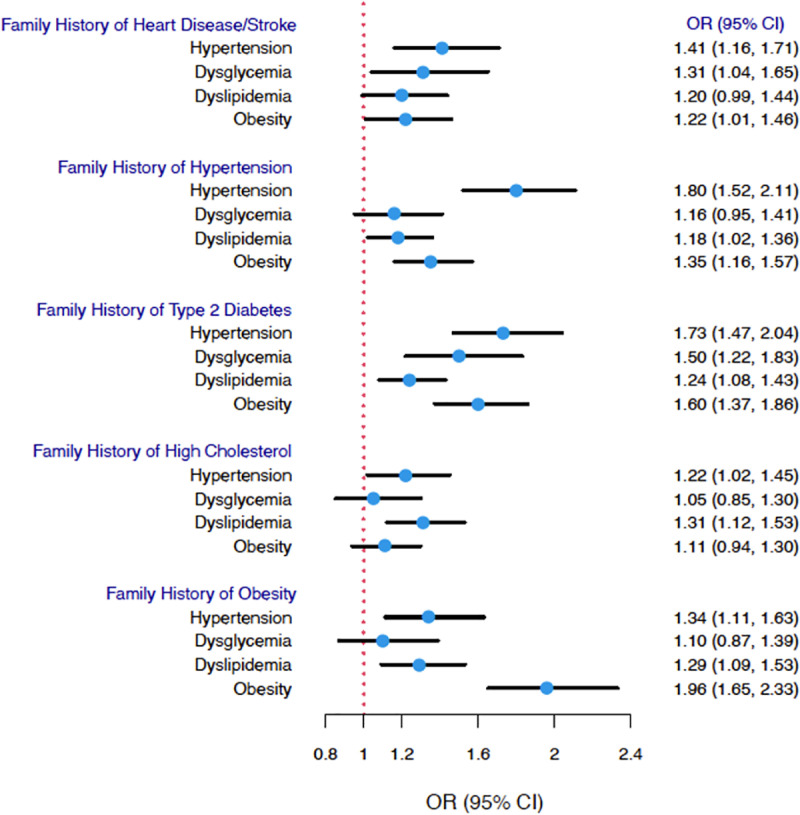
Association between family history of disease and the risk of cardiometabolic risk factors.

Fig 1 illustrates the association of having a positive family history of disease with each CRF. A positive family history of a specific disorder had the highest association with the same disorder in offspring. For example, having a family history of hypertension was associated with increased odds of developing hypertension (OR 1.80 (CI 95% 1.52 – 2.04)), while a positive family history of obesity doubled the odds of having obesity (OR 1.96. (95% CI: 1.65 – 2.33)). On the other hand, a positive family history of T2D was significantly associated with increased odds of all CRFs.

Multivariate Logistic regression models with family history as predictor adjusted for age and sex.

### Association between the categories of family history of each disease and the corresponding CRF

**Fig 2:**
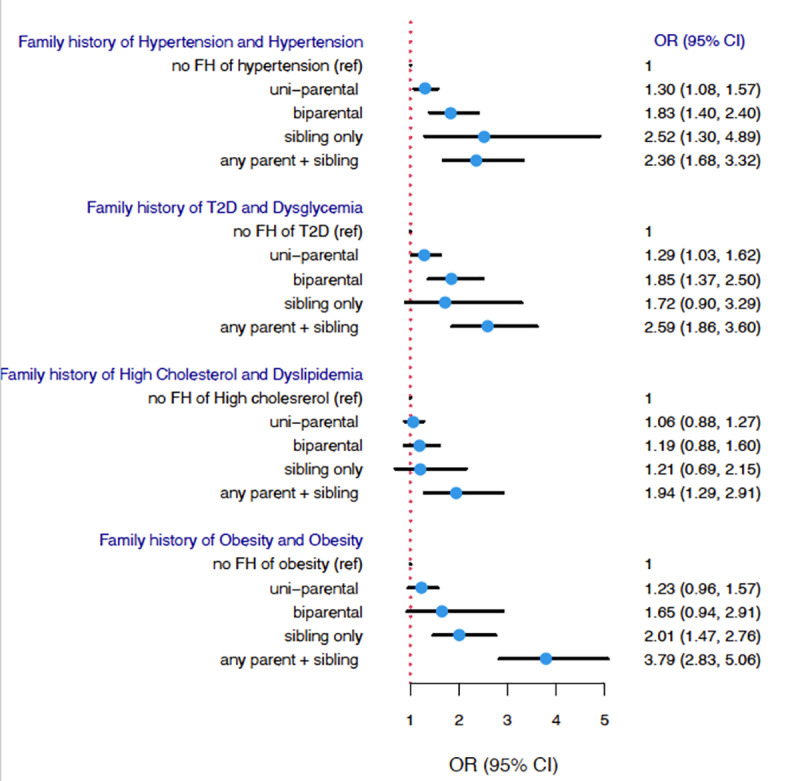
Association between the categories of family history of each disease and corresponding CRF.

Fig 2 shows the associations between the levels of family history of a specific disorder and their relationship with the disorder or CRF. The odds of CRFs increased as family members with the disease increased. This pattern was observed for dysglycemia, dyslipidemia and obesity. Hypertension exhibited the strongest association with sibling history of hypertension.

Multivariate Logistic regression models adjusted for age, gender, and all types family history of diseases.

## Discussion

CRFs are highly prevalent in our sample, with a quarter of adults classified as obese, a fifth had hypertension, over half had dyslipidemia, and more than one in ten adults had dysglycemia (Table 1). More than half of our study population reported having a family history of obesity, dyslipidemia, hypertension, T2D, and heart disease/stroke.

In our study, we observed that individuals with a positive family history had a significantly higher prevalence of CRFs compared to those who reported no family history in our sample. These findings align with the findings from the National Health and Nutrition Examination Survey (NHANES) cohort in the United States, where healthy respondents with a family history of CVD exhibited significantly higher percentages of poorer health metrics such as increased BMI, cholesterol, blood pressure, and glycemic markers [4].

Table 2 provides the age-adjusted prevalence of CRFs according to each family history of disease. The most frequently reported family history of disease among our study population was T2D, followed by hypertension. Our sample is a young population (mean age was 25.7 ± 6.1 years); therefore, we assume that their parents are also young to have reported heart disease/stroke, hence the 16.2%.

Similar to a previous study that differentiated between parental and sibling family histories [27], we categorized the family history components into five subcategories; no family history, uniparental – reporting only one parent having disease, biparental – reporting both parents with the disease, sibling only – having sibling(s) with the disease without parents, and having any parent and sibling(s) combined (Table 2).

There was a consistent trend of increasing CRF proportions as the number of relatives with the disease increased. This trend is presented for the prevalence of hypertension, dysglycemia and obesity, where they were highest in the 4^th^ category of family history; any parent plus sibling. For example, we found that in respondents that had a family history of hypertension, the prevalence of hypertension significantly increased in those that had a family history from one parent only compared with those that had a family history from parent(s) and sibling(s) combined. This is in line with the findings from the study led by Ranasinghe et al. [12], which demonstrated that the prevalence of hypertension increased with the number of generations affected by hypertension.

Fig 1 represents an illustration of the association between each family history of disease and CRFs. We found that a positive family history of any of the diseases examined consistently affected the odds of developing hypertension in offspring. The odds have increased by 41%, 80% and 73% in individuals with family histories of heart disease/stroke, hypertension and T2D, respectively.

Interestingly, our study revealed that having a family history of T2D significantly increased the odds of developing all CRFs in our sample. This finding aligns with the study in Ghana led by Lokpo et al. [28], which demonstrated a significant association between a history of diabetes and metabolic syndrome, encompassing hypertension, abdominal obesity, dysglycemia, and hyperlipidemia. Another study in the United States confirmed that family history of T2D is associated with obesity and hypertension [29]. This can be explained by the pathophysiological pathway described by Chakraboty et al. [30]. Diabetes and insulin resistance cause a disruption in the regulation of lipogenesis; causing an increase in Triglycerides and LDL-cholesterol and lowering HDL-cholesterol. The increase in lipid molecules exacerbates inflammation and plaque formation. Together, these factors contribute to the emergence of hypertension.

The CRFs increased considerably in relation to their corresponding specific history of disease. For instance, in the family history of high blood pressure group, the prevalence of hypertension doubled while other CRFs showed only modest changes. The prevalence of dysglycemia increased to 21.5% in individuals with diabetic parents and siblings. Similarly, the prevalence of obesity doubled to 53.5% in those with parental and sibling family history of obesity (Table 2).

Moreover, Fig 2 displayed the association between the four categories of family history of a specific disorder and the corresponding disorder itself, after adjusting for all types of family histories. We found that a positive family history increased the odds of having the disorder from one parent, two parents, to parent plus sibling combined, indicating a dose-response trend. This trend was observed for every CRF analyzed. This finding is consistent with the findings from the Lifelines Cohort Study [31], where they demonstrated the likelihood of an individual with a first-degree relative with a cardiometabolic disorder had a higher risk of developing the same disorder, where the risk increased by 23% for hypertension and by 2.48-fold for T2D.

The principal strengths of this investigation include the large sample size and the depth of information included in the study. The ample sample size allowed for robust statistical analysis and reliable findings. Moreover, we were able to investigate the different types of family histories of diseases and the number of affected family members. This comprehensive approach provided valuable insights into the influence of having one or both parents, as well as sibling(s) affected with CVD-related diseases on the individual’s health.

Our primary limitation of this study is the nature of the sample, which is volunteer-based and therefore introduces selection bias, potentially affecting the representativeness of the study, and therefore its generalizability. Moreover, the high percentage of missing data for behavioral factors such as smoking and physical activity did not allow us to include them in the regression models. Family history of disease was self-reported in our study and therefore introduces recall bias. Furthermore, we assume that because our population was very young (mean age 25.7 ± 6.1 years), the prevalence of positive family history of CVD and its risk factors may have been underestimated.

In future analyses, it would be beneficial to include additional information regarding the parental history of CVD, specifically distinguishing between maternal and paternal history, as well as considering the age at diagnosis. Previous research has demonstrated that these factors can have differential effects on the offspring’s health risk. For example, findings from the Framingham study revealed that fathers diagnosed with CVD before the age of 55 years and mothers diagnosed before the age of 65 years increased the risk in offspring by 75% and 60%, respectively [2,13].

Furthermore, parental consanguinity information can potentially enrich the study’s outcome. Among Emiratis, consanguineous marriages are highly prevalent ranging from 33% and 65% depending on first-degree or distant relationship, and the rate can be higher in rural areas [32, 33]. Studies that have included consanguinity, or in-breeding, in CVD risk showed that the magnitude of the CVD risk was higher among offspring of consanguineous couples. They reported a 2.62-fold increase in the risk for developing heart disease, 2.44-fold increase for T2D, and 2.62-fold increase for hypertension, compared to offspring of non-consanguineous couples [34, 35]. Additionally, a Saudi study showed that off-springs of first-cousin couples were three times more likely to develop obesity [36].

In summary, our study confirms the strong association between family history of cardiovascular disease (CVD) and its risk factors with cardiometabolic risk factors (CRFs) in offspring. It is crucial to incorporate family history assessment and consider the number of affected relatives during health screening procedures. This approach allows for the early identification of individuals, including asymptomatic young adults, who might be at an elevated risk of developing CVD. Such individuals can potentially benefit from personalized health messages that emphasize lifestyle modifications, regular monitoring, and, when necessary, medication to manage cardiometabolic markers [37]. By implementing these strategies, we can effectively target and mitigate CVD risk in higher-risk individuals, ultimately improving long-term cardiovascular health outcomes.

## Abbreviations

BMI: Body Mass Index
Chol: Cholesterol
CRFs: Cardiometabolic risk factors
CVD: Cardiovascular disease
CAD: Coronary artery disease
FBG: Fasting blood glucose
FH: Family history
HbA1c: Hemoglobin A1C
HDL: High density lipoprotein
IDF: International Diabetes Federation
LDL: Low density lipoprotein
METs: Metabolic equivalents
NCD: Noncommunicable disease
SES: Socioeconomic status
TG: Triglycerides
UAE: United Arab Emirates
UAEHFS: UAE Healthy Future Study
